# Blood cancer prediction using leukemia microarray gene data and hybrid logistic vector trees model

**DOI:** 10.1038/s41598-022-04835-6

**Published:** 2022-01-19

**Authors:** Vaibhav Rupapara, Furqan Rustam, Wajdi Aljedaani, Hina Fatima Shahzad, Ernesto Lee, Imran Ashraf

**Affiliations:** 1 School of Computing and Information Sciences, Florida International University, University Park, USA; 2 Department of Computer Science, Khwaja Fareed University of Engineering and Information Technology, Rahim Yar Khan, 64200 Pakistan; 3 Department of Computer Science and Engineering, University of North Texas, Denton, TX USA; 4 Department of Computer Science, Broward College, Broward County, FL USA; 5 Department of Information and Communication Engineering, Yeungnam University, Gyeongsan, 38544 South Korea

**Keywords:** Cancer, Diseases

## Abstract

Blood cancer has been a growing concern during the last decade and requires early diagnosis to start proper treatment. The diagnosis process is costly and time-consuming involving medical experts and several tests. Thus, an automatic diagnosis system for its accurate prediction is of significant importance. Diagnosis of blood cancer using leukemia microarray gene data and machine learning approach has become an important medical research today. Despite research efforts, desired accuracy and efficiency necessitate further enhancements. This study proposes an approach for blood cancer disease prediction using the supervised machine learning approach. For the current study, the leukemia microarray gene dataset containing 22,283 genes, is used. ADASYN resampling and Chi-squared (Chi2) features selection techniques are used to resolve imbalanced and high-dimensional dataset problems. ADASYN generates artificial data to make the dataset balanced for each target class, and Chi2 selects the best features out of 22,283 to train learning models. For classification, a hybrid logistics vector trees classifier (LVTrees) is proposed which utilizes logistic regression, support vector classifier, and extra tree classifier. Besides extensive experiments on the datasets, performance comparison with the state-of-the-art methods has been made for determining the significance of the proposed approach. LVTrees outperform all other models with ADASYN and Chi2 techniques with a significant 100% accuracy. Further, a statistical significance *T*-test is also performed to show the efficacy of the proposed approach. Results using k-fold cross-validation prove the supremacy of the proposed model.

## Introduction

Cancer is the abandoned outgrowth of abnormal cells that may spread to different parts of the human body^1^. Currently, it is one of the leading causes of death in the world. Study^2^ shows that approximately 10 million cancer deaths and 19.3 million new cases appeared only in 2020. The mortality rates of the different types of cancer vary concerning the type of cancer. For example, in 2020, lung cancer has 18%, colorectal cancer has 9.4%, while liver cancer, stomach cancer, and breast cancer has mortality rates of 8.3%, 7.7%, and 6.9%, respectively. Blood cancer constitutes nearly 10% of all the newly diagnosed cancer cases^1^. Early diagnosis and prediction have been considered prudent ways to reduce cancer deaths worldwide.

In this regard, this study focuses on the prediction of blood cancer. As noted by the Leukemia and Lymphoma Society^3^, in the United States (US) alone, 1,290,773 people have blood cancer. The common types of blood cancers include myeloma, leukemia, lymphoma, myelodysplastic syndromes, among others. To be discrete, blood cancers affect the blood cells, bone marrow, lymph nodes, as well as other parts of the lymphatic system. Currently, research has led to the development of therapies that improve the immunity system of affected individuals so that they can deal with cancer cells.

Previous studies on blood cancer prediction have utilized different models and algorithms for predicting blood cancer, which yielded various accuracy and precision levels. For example, Goutam et al.^4^ utilized support vector machines (SVM) to achieve a precision of 85.74%, specificity of 80%, and sensitivity of 100%. Study^5^ used H20 deep learning and got an accuracy of 79.45%. Additionally, Vijayarani and Sudha^6^ applied K Means, Fuzzy Means, and Weighted K Means which achieved an accuracy of 78%, 75%, and 85%, respectively. Similarly, Xiao et al.^7^ used k-nearest neighbor (KNN), SVM, decision trees (DT), random forest (RF), and gradient boosting decision trees to achieve accuracy of 99.20%, 98.78%, and 98.41%, respectively. On the other hand, Subhan et al.^8^ leveraged KNN and Hough transform to obtain an accuracy of 93%. Gal et al.^9^ used KNN, SVM, and RF classifiers for achieving accuracy scores of 84%, 74%, and 81%, respectively. Despite such efforts to elevate the performance of the machine and deep learning classifiers, the desired accuracy is not met for blood cancer prediction.

The chief objective of the current study is to propose an approach that can perform blood cancer prediction with high accuracy using microarray gene data. Of the challenges associated with this task, the data imbalance and the high dimensionality of data are two important problems. To overcome these issues, the current study uses adaptive synthetic (ADASYN) oversampling and Chi-square (Chi2). In summary, this study makes the following contributions

- The performance of well-known machine learning algorithms is analyzed on microarray gene data. These algorithms include RF, logistic regression (LR), support vector classifier (SVC), KNN, Naive Bayes (NB), extra tree classifier (ETC), DT, and Adaboost classifier (ADA).
- A hybrid model called LVTrees is proposed which utilizes RL, SVC, and ETC through the majority voting. For data balancing the influence of ADASYN is investigated while Chi2 is used to select the optimal set of features for classification.
- Extensive experiments are conducted to evaluate the efficacy of the proposed approach. In addition, several state-of-the-art methods are compared with the proposed approach. The statistical significance test is also performed to analyze the validity of the proposed approach. Results are further validated using k-fold cross-validation.

The rest of the paper is organized as follows. The following section discusses the research papers related to the current study. The proposed methodology is described in the section “Materials and methods” while the section “Results and discussions” contains the analysis and discussion of results. In the end, the “Conclusion” section concludes the paper and highlights the direction for future work.

## Related work

Owing to the importance of the healthcare domain, several research works can be found in the literature that focus on cancer prediction using machine and deep learning approaches. For example, studies^10,11^ perform cancer prediction using image-based approaches. Similarly, Goutam et al.^4^ developed an automated system for the diagnosis of leukemia. The framework supports a variety of strategies like K-means clustering etc. The data are obtained from hospitals for examining the performance of the proposed method as a binary classifier. Results show that it obtains a 98% accuracy for cancer prediction. While Vijayarani and Sudha^6^ focused on the prediction of disease using hemogram blood test data. A new algorithm called weight-based K-means is proposed to diagnose various diseases, e.g., human immunodeficiency virus (HIV) and viral infection. Tests are performed on data from 524 patients, and results show that the proposed algorithm achieves significantly higher accuracy than the Fuzzy C-methods and K-means clustering algorithms.

In the same way, a multi-model ensemble is presented in^7^ for predicting cancer. The authors analyzed the gene data gathered from the stomach, breast, and lung tissues. The DESeq approach is used to avoid overfitting in classification which helped identify genetic details differentiated between normal and tumor phenotypes. Moreover, it controlled the dimensionality of data and enhanced the forecast accuracy along with the significant reduction in computational time. Study^12^ developed an automated method of detecting and classifying acute lymphoblastic leukemia based on a deep convolutional neural network (CNN). To test the performance, comparisons are made with different color models. The results show that the proposed method achieved high accuracy without requiring microscopic image segmentation. The authors presented a diagnosing method in^13^ to predict the primary stage of cancer. The model is integrated between hybrid feature selection and preprocessing phases. From a subset of 25 features, the proposed model showed the highest accuracy with 14 optimal features. A four-phase process is employed to train the subset of the optimal feature. Results show that the classification accuracy can be greatly improved by using preprocessing methods and feature selection before selecting the data.

Study^14^ proposed classification models to distinguish the blood microscopic images of patients affected by leukemia from those free of leukemia. To extract the features, a pre-trained CNN name AlexNet and various other classifiers are used. Tests show that SVM got better results compared to other classifiers. In the second model, extraction and classification are done using AlexNet only where results show its superiority over other models concerning different performance metrics.

**Table 1:** Summary of the systematic analysis studies in related work.

Study	Models	Dataset	Evaluation metrics	Results
^15^	Bayes Network learning, Conjunctive Rule, NBTree, VFI, Random Subspace, Naïve Bayes Updateable, and PART	Three datasets contains 7130 Genes	Accuracy	97.22% for 500 genes
^4^	Local Directional path	90 high-quality $184 \times 138$ size images obtained from the American Society of Hematology	Sensitivity, Specificity, Precision, F-Measure	Sensitivity: 100%, Specificity: 80%, Precision: 85.74%, F-Measure: 93.4%
^6^	K-Means, Fuzzy C Means, Weighted K Means	Heart dataset from UCI machine learning repository	Cluster accuracy, error rate and execution time	Leukemia, K-Means: 78%, Fuzzy means: 75%, WK-Means: 85%
^13^	Updatable NB, MLP, KNN, SVM	25 variables or features and 82 instances or records	Accuracy	NB 94.76%, MLP 95.24%, SVM 96.20%, KNN 91.43%
^16^	Fuzzy c-means clustering, PCA, SVM	21 peripheral blood smear and bone marrow slides of 14 patients with all and 7 normal persons $2592 \times 3872$ pixels in red green blue (RGB) color	sensitivity, specificity, accuracy, precision and false negative	Sensitivity 98%, Specificity 97%, Accuracy 98%, Precision 98%
^17^	Linde–Buzo–Gray, Kekre’s Propotionate Error, K-Means	115 digital images of size $256 \times 256$. 16 datasets with 2415 images, 642 images with size $632 \times 480$ pixels	Sensitivity, specificity, accuracy	Sensitivity 100%, Specificity 99.747%, Accuracy 99.7617%
^7^	KNN, SVM, DT, RF, GBDT	Three RNA-seq data sets	Precision, recall and accuracy	Accuracy LUAD: 98.80 (± 1.79), STAD: 98.78 (± 1.44), BRCA: 98.41 (± 0.41)
^12^	Deep convolutional neural networks	Images from ALL-Image DataBase (IDB)	Sensitivity, specificity, accuracy	Sensitivity 100%, Specificity 98.11%, Accuracy of 99.50%
^14^	AlexNet	2,820 images	Precision, Recall, accuracy	100% classification accuracy
^18^	Alert Net-RWD	16 datasets with 2,415 images	Accuracy, precision	Accuracy 97.18%, Precision 97.23%
^19^	SVM, KNN, NB, and RF	NCBI/GEO public database: 11 series from Microarray and 2 series from RNA-seq	ANOVA statistical test, accuracy, F1	10 Genes F1-score: SVM: 97.13%, KNN: 96.28%, NB: 97.29%, RF: 97.01%
^20^	DNN deep learning network	36 cases containing 22,283 gene expression of acute myeloid leukemia (AML) microarray	Accuracy	Accuracy: 96.6%

A study^20^, very similar to ours, used the Leukemia_GSE9476 dataset^21^ with a deep learning approach to analyze its diagnostic performance compared to traditional methods. The study used Leukemia microarray gene data which consists of 22,283 genes. Normalization tests are used at preprocessing stage while a DNN neural network is used for training and testing. Experimental results indicate that using the traditional method achieved an accuracy of 0.63, whereas the deep learning network achieved an accuracy of 0.96%. Another study^19^, used the Leukemia_GSE28497 dataset^22^ to study the integration of multiple microarrays and ribonucleic acid (RNA)-seq platforms. Four types of Leukemia samples are analyzed in the study. The minimum redundancy maximum relevance (mRMR) is used for feature selection. Results show that 96% accuracy can be achieved using only a small portion of ten genes. Analysis of variance (ANOVA) statistical test is performed to verify the performance of the model for multi-class classification.

For improving the Leukemia classification process, Abd El-Nasser et al.^15^ proposed an enhanced classification algorithm (ECA) using the select most informative genes (SMIG) module and a standardization process. Evaluation results showed that the proposed ECA system achieves 98% accuracy in 0.1s time when preprocessing and classification are done. Compared to the methods used in previous studies, the proposed system achieved better results. The authors propose an automatic diagnostic method in^18^ to predict acute myeloid and acute lymphoid Leukemia. The study utilizes a CNN model called Acute Leukemias Recognition Network - Residual Without Dropout (Alert Net- RWD) for this purpose. In the Alert Net-RWD model, the Alert Net part consists of five convolutional layers, batch normalization, and max-pooling layers. The residual layer without dropout is followed by the max-pooling layers in the Alert Net-RWD model. Compared to other CNN architectures, Alert Net-RWD uses fewer parameters. Test results show that the proposed model achieves 97.18% accuracy and 97.23% precision. Study^17^ proposed an algorithm for the detection of blast cells under specific criteria of image enhancement and processing. It comprises a selection of the panel, use of K-means clustering for segmentation, followed by a refinement process. A public database is used for testing, and the results show that the proposed algorithm achieves 97.47% sensitivity and 98.1% specificity. Another dataset collected from local hospitals is also used for experimental purposes which shows that the algorithm led to 100% sensitivity, 99.747% accuracy, and 99.7617% accuracy. In a similar fashion, an enhanced computer-based method for cancer cell prediction is introduced in^16^. The authors use principal component analysis (PCA)-based features extracted from the nucleus image of these cells. In addition to detecting cancerous cell subtypes, the proposed algorithm can differentiate non-cancerous cell subtypes with improved sensitivity.

Despite the tremendous results reported in the above-mentioned studies, using microarray gene data is not very well studied concerning blood cancer prediction. Besides, apart from a couple of research works, the accuracy reported in the rest of the research works is not sufficient enough for the blood cancer prediction. In addition, predominantly, research works use smaller datasets and results cannot be generalized. To overcome such limitations, this study proposes a hybrid model to achieve higher accuracy for blood cancer prediction. Table 1 summarizes the systematic analysis studies in related work.

## Materials and methods

This section contains details about used datasets, techniques, and methods for the blood cancer prediction experiment.

### Proposed approach overview

**Figure 1:**
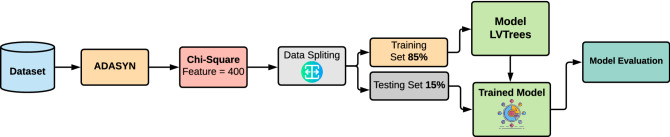
Methodology applied for the study.

Figure 1 shows the architecture of the proposed methodology. It consists of feature selection, data resampling, data splitting, model training, model evaluation.

First, the dataset is extracted from the National Center for Biotechnology Information (NCBI) https://www.ncbi.nlm.nih.gov/. Afterward, preprocessing is done on the dataset to improve the efficiency of learning models. In preprocessing, data resampling and feature selection are performed. Data resampling aims at dataset balancing by generating data for minority classes and the ADASYN technique is used for that purpose^23^. The number of samples for each target class after applying ADASYN is shown in Table 2.

**Table 2:** Number of samples for each class with and without applying ADASYN technique.

Target	Count	After ADASYN
B-CELL_ALL	74	74
B-CELL_ALL_TCF3-PBX1	22	74
B-CELL_ALL_HYPERDIP	51	64
B-CELL_ALL_HYPO	18	74
B-CELL_ALL_MLL	17	73
B-CELL_ALL_T-ALL	46	74
B-CELL_ALL_ETV6-RUNX1	53	76
Total Samples	281	509

Because of the large feature set, feature selection is performed^24^ to reduce complexity for the training data. Important features are selected using Chi2 feature selection techniques^25^. Chi2 selects the best 400 gene features for the excellent fit of learning models as shown in Table 3. The selection of the number of features for experiments is based on empirical findings.

**Table 3:** Number of features for experiments.

Features	Original	After Chi2
Total	22,283	400

Preprocessing makes the dataset ready for the training of learning models. The data are split into training and testing parts with an 85:15 ratio, respectively, as shown in Table 4. The 85% data are used for the training of learning models because the overall size of the dataset is not large enough. It provides enough training data for the learning models to have a good fit. After training, 15% data are for the evaluation of the trained model using accuracy, precision, recall, and F1 score.

**Table 4:** Number of samples and features in training and testing sets.

Techniques	Training set		Testing set	
	Samples	Features	Samples	Features
Original dataset	238	22,283	43	22,283
After ADASYN	432	22,283	77	22,283
After Chi2	238	400	43	400
After ADASYN+Chi2	432	400	77	400

### Data description

Results are predicted using the Leukemia_GSE28497 dataset^22^. The dataset contains 281 samples, 22,285 genes (features), and seven target classes as shown in Table 5 and a sample of dataset shown in Table 6. Table 6 contains the samples of the original data using the microarray gene technique. ‘Type’ attribute shows the type of blood cancer while other columns are the gene properties that can be used to differentiate the patients and healthy people. The values of these gene properties are determined using the microarray test. Every single row indicates one sample of blood monitored by the microarray.

**Table 5:** Target count for each class in Leukemia_GSE28497 dataset.

Target	Count
B-CELL_ALL	74
B-CELL_ALL_TCF3-PBX1	22
B-CELL_ALL_HYPERDIP	51
B-CELL_ALL_HYPO	18
B-CELL_ALL_MLL	17
B-CELL_ALL_T-ALL	46
B-CELL_ALL_ETV6-RUNX1	53
Total Samples	281

**Table 6:** Sample of Leukemia_GSE28497 dataset.

Type	1007_s_at	1053_at	.	AFFXTrpnXM_at
BCELL_ALL	7.409521	5.009216	.	2.608381
BCELL_ALL	7.177109	5.415108	.	2.634063

To further validate the proposed model, this study uses an additional dataset called Leukemia_GSE9476^21^. The dataset contains 64 samples as shown in Table 7. The sample of the validation dataset is shown in Table 8.

**Table 7:** Target count for each class in Leukemia_GSE9476.

Target	Count
AML	26
Bone_Marrow	10
Bone_Marrow_CD34	8
PB	10
PBSC_CD34	10
Total	64

**Table 8:** Sample of Leukemia_GSE9476 dataset.

Type	1007_s_at	1053_at	.	AFFXTrpnXM_at
Bone_Marrow_CD34	7.745245	7.811210	.	4.139249
Bone_Marrow_CD34	8.087252	7.240673	.	4.122700

### Supervised machine learning models

This study uses different machine learning models for blood cancer prediction such as RF, LR, SVC, KNN, NB, ETC, DT, ADA, and proposed LVTrees. Table 10 presents the description of the machine learning models used in this study, while the hyperparameter setting for all machine learning models is shown in Table 9. Optimal parameters are obtained using the grid search method where each parameter from the given range is executed against the input data to analyze a model’s performance. This process is repeated for each parameter and finally, the parameters’ values for optimal performance are obtained.

**Table 9:** Models hyperparameters settings and hyperparameter range used for tuning.

Model	Hyperparameters setting	Hyperparameter range
RF	n_estimators = 300, max_depth = 25	n_estimators = 20 to 500, max_depth = 2 to 50
LR	multi_class = “multinomial”, C = 2.0	solver = liblinear,saga sag, multi_class = “multinomial”, C = 1.0–5.0
SVC	kernel = “linear”, C = 2.0	kernel = linear, sigmoid, poly, C = 1.0–5.0
KNN	n_neighbors = 4	n_neighbors = 2–6
NB	Default setting	–
ETC	n_estimators = 300, max_depth = 25	n_estimators = 20–500, max_depth = 2–50
DT	max_depth = 25	max_depth = 2–50
ADA	n_estimators = 300, learning_rate = 0.2	n_estimators = 20–500, learning_rate = 0.1–0.8
LVTrees	Model (LR, SVC,ETC), Voting = Hard	Voting = Hard and Soft

**Table 10:** Description of used machine learning models.

Model	Description
RF	RF is a model for tree-based ensemble learning that predicts accurately by combining multiple poor learners. IT uses the bagging method for training several decision trees with different samples of bootstrap. The substitution of training data in random forests is a bootstrap study, where the sample is the same as the training collection^26^
LR	The classification problems are generally dealt with using logistic regression. It is a regression model based on the probability theorem and a predictive analysis algorithm. Binary information, in which one or more variables work together to generate a result, is most often interpreted. Using the sigmoid logistic regression function, a relationship is established between one or more independent variables with an approximation probability^27^
SVC	The classification aims to divide a data collection into categories based on a set of criteria to classify data in a more meaningful way. SVC is a classification method focused on the support vector technique. The SVC’s goal is to fit the data you supply and return a “best fit” hyperplane that separates or categorizes the data. Following that, you should feed any features to your classifier to see what the “predicted” class is after you have obtained the hyperplane. This makes this algorithm particularly good for our purposes, though it can be used in a variety of contexts^28,29^
KNN	KNN is a basic model used in machine learning for regression and classification processing. The data is referred to as the class with the closest neighbors, and the technique uses the data to organize the current data means based on a distance attribute. The KNN model bestows pledge effects in this experiment when the value of *k* is equal to five (k = 5). It means it looks at the five closest neighbors and chooses one based on the majority or closest distance^30^
NB	Focused on the Bayes Theorem, the controlled learning algorithm called the Naive Bayes algorithm is used to resolve classification problems. The training of an NB classifier involves a limited number of data points and is therefore fast and scalable. It is a probabilistic classifier that predicts the probability of an object. The NB classifier claims that each likelihood of feature is independent of the others and that they do not overlap, such that each feature contributes similarly to a sample belonging to a given class. The NB classifier is easy to use and quick to compute, and it works well on massive datasets of high dimensionality^31^
ETC	The ETC works in a similar way to the random forest, except for the process of tree building in the forest. The ETC uses the initial training sample to build each decision tree. The top function to interrupt the data in the tree is chosen using the Gini index, and *k* samples of the best functions are used to make the decision. Several de-correlated decision trees were developed using these random function indicator samples. The algorithm for decision trees is an algorithm for categorical and numerical data that works perfectly^32^
DT	A DT is a kind of tree-like framework used to construct structures. A decision tree is commonly used in medical processing because it is quick and fast to execute. There are three nodes in the decision tree. (1) Root node (main node; other nodes’ roles are dependent on it); Interior node (it handles various types of attributes) (3) Node of the leaf (it is also called as end-node; it is the final node which represents the results of each test)^33^
ADA	ADA is typically used in combination with other algorithms to improve their accuracy. It focuses on boosting vulnerable learners into good learners. Any AdaBoost tree is based on an error rate of the last constructed tree^34^

### Proposed model LVTrees

**Figure 2:**
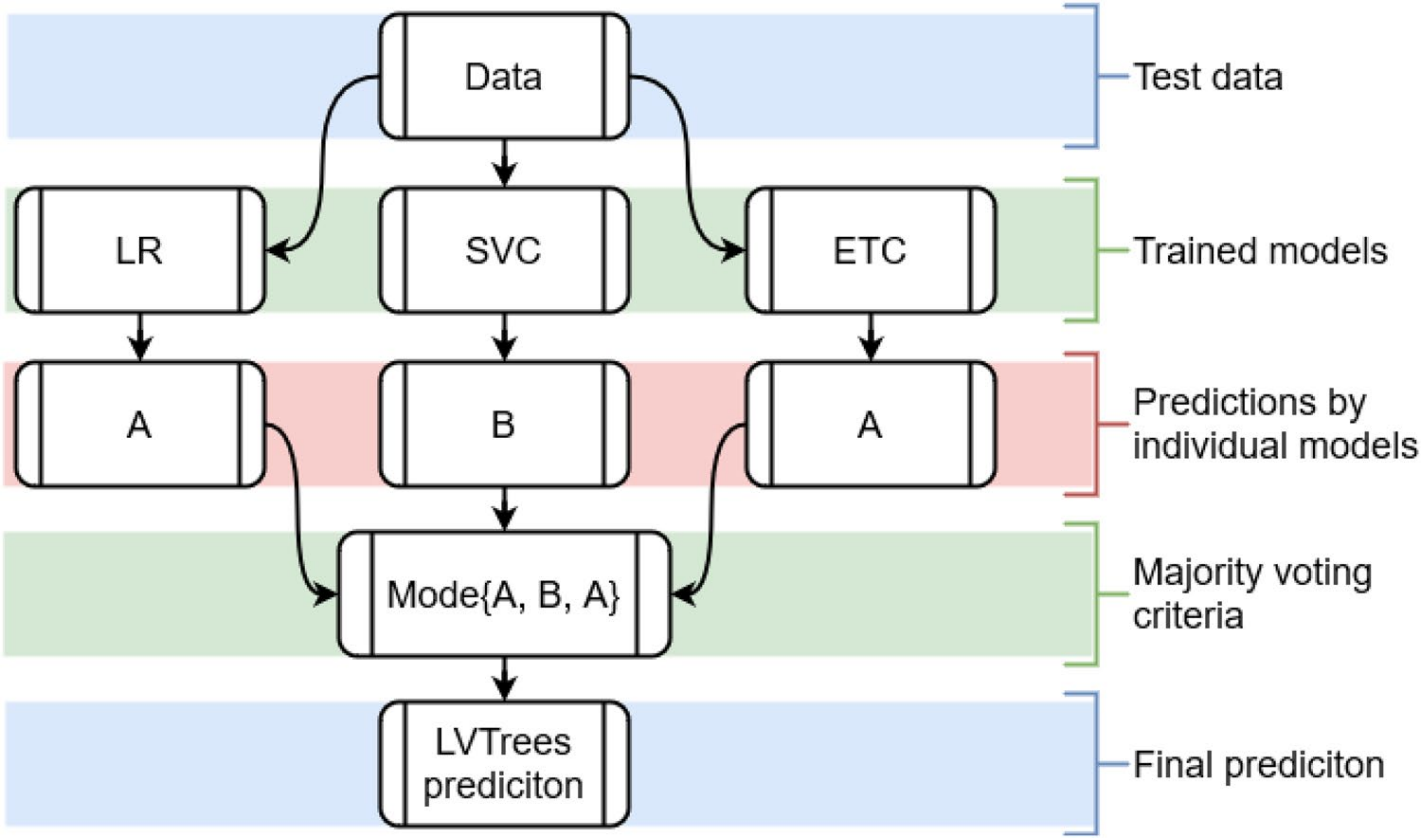
Architecture of proposed hybrid LVTrees model.

This study proposes a hybrid model called LVTrees, which combines three models including LR, SVC, and ETC. These models are selected for ensemble based on their performances on the used blood cancer dataset. Models are combined using a majority voting criterion, which indicates that the most predicted class by the models will be considered the final prediction by LVTrees. Let *A*, and *B* be two target classes in the dataset, out of three models in LVTrees *A* be the prediction from two models and *B* be the prediction of one model, then the final prediction will be class *A* under the majority voting. LVTrees’ deep hybrid architecture makes it significant for blood cancer prediction. The algorithm of the proposed LVTrees model is shown in Algorithm 1 and architecture is shown in Fig. 2. 
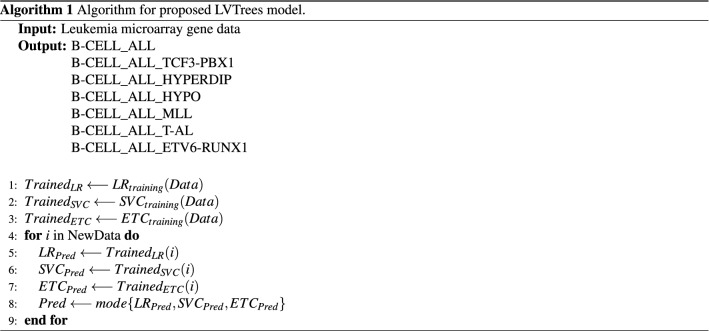

### Chi-square (Chi2)

The Chi2 is the most frequently used feature selection method in machine learning^35^. In the current study, it is used to select the best features for the training of models. The used datasets for the experiment contain a large feature set of 22,283 features. The large feature set can create complexity in the learning procedure of models. As a result, only the best features selected using Chi2 are used to improve the performance of machine learning models. Equation 2 is used by Chi2 to calculate the score:

1 $$\begin{aligned} X^2 (D,t,c)= \sum _{e_t \epsilon \{0,1\}} \sum _{e_c \epsilon \{0,1\}} \frac{(N_{e_t,e_c} - E_{e_t,e_c})^2}{E_{e_t,e_c}} \end{aligned}$$

where, *N* is the frequency observed and *E* is the frequency predicted, $e\_t$ is given the value 1 where *t* and 0 are contained in this text, while $e\_c$ takes 1 if the document is in *c* class and 0 otherwise.

A high Chi2 score for each feature indicates that the null hypothesis $H_0$ of independence (that the document class has no impact on the term’s frequency) should be disregarded, and the feature and class are interdependent. In this case, the microarray gene feature should be selected for model training.

### ADASYN resampling

Imbalanced learning happens when one or more classes in the training dataset have a substantially lower number of samples than others. It means that the data distribution of one class significantly dominates the instance space of others. Under-sampling and oversampling are two standard approaches for resolving this problem. For under-sampling, the data from the dominant class is discarded to equal the samples of dominant and non-dominant groups. However, this is a massive waste of information. The discarded data could contain crucial information about the majority class. Over-sampling is the process of generating samples for the minority class until it is the same size as the majority class. Since most of the scenarios have already been used repetitively, the model would be too focused on a minority of the cases, which is the most common error in model building. ADASYN can be used to prevent any of the problems listed above. ADASYN is a synthetic data generation algorithm with the main benefits of not copying minority data and producing more data for ’harder to understand’ scenarios. It follows these steps

- Using the formula $d = \frac{m_s}{m_l}$ , calculate the ratio of minorities to majorities. The numbers $m_s$ and $m_l$ represent the number of minority and majority class examples, respectively. Start the algorithm if *d* is less than a certain value.
- Calculate the total sum of synthetic minority data to be generated using $G = (m_l - m_s) \beta$. *G* stands for the total number of minorities for which data is needed. Following ADASYN, is the best minority-to-majority data ratio. The meaning $\beta = 1$ after ADASYN signifies a perfectly balanced data set.
- Locate each minority example’s KNN and calculate the $r_i$ value using $\frac{Number\, of\, majority}{k}$. After that, each minority case study should be assigned to a specific neighborhood. The $r_i$ definition denotes a neighborhood’s dominance by the dominant class.
- Normalize the $r_i$ values such that the total number of $r_i$ values equal 1.
  $$\begin{aligned}&\hat{r_i} = \frac{r_i}{\sum {r_i}} \\&\sum \hat{r_i} = 1 \end{aligned}$$
- Using the formula, calculate the number of synthetic examples to obtain per neighborhood.
  $$\begin{aligned} {G_i} = G{\hat{r_i}} \end{aligned}$$
- $G_i$ data for each neighborhood should be generated using:
  $$\begin{aligned} {s_i} = {x_i}+({x_{zi}} - {x_i})\lambda \end{aligned}$$

## Results and discussions

All the experiments are performed on the Core i7 7th generation machine with the window 10 operating system. Machine learning models are implemented using the sci-kit learn, pandas library, and Python language on the Jupyter notebook.

### Models performance on original leukemia dataset

**Figure 3:**
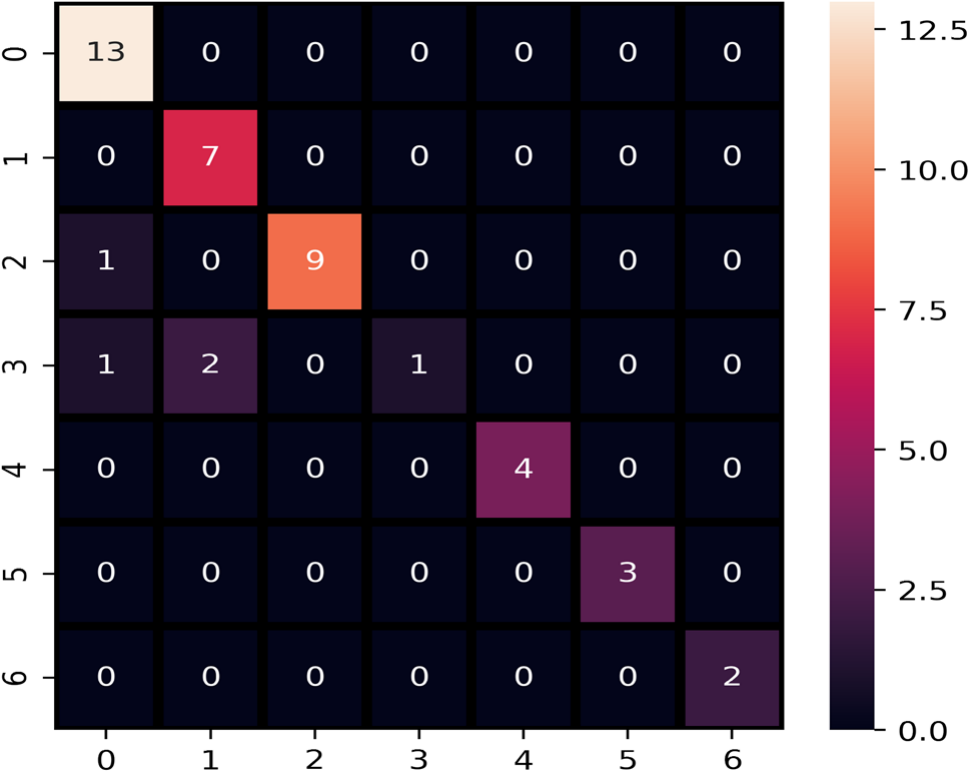
Confusion matrix of LVTrees on original dataset.

Initially, the performance of machine learning is evaluated on the original blood cancer dataset, and the results are presented in Table 11. On the original dataset, LR, KNN, and LVTrees models outperform all other models in terms of accuracy score. These models achieve the highest accuracy of 0.91 on the original dataset, while the proposed model LVTrees outperform all other models concerning the recall and F1 score. LVTrees achieves the highest recall and F1 scores of 0.89 on the original dataset showing its significance for blood cancer prediction. This significant performance of LVTrees is because of its hybrid architecture, which is a combination of linear and tree-based models. On the other hand, LR and KNN also perform well and show equal accuracy to LVTrees. ADA performs poorly because of the small size of the dataset because it requires a large number of records to boost its accuracy due to its boosting approach. Figure 3 shows the confusion matrix of best performer LVTrees on the original dataset which shows that LVTrees gives 39 correct predictions out of a total of 43 predictions. In confusion matrices, B-CELL_ALL, B-CELL_ALL_ETV6-RUNX1, B-CELL_ALL_HYPERDIP, B-CELL_ALL_HYPO, B-CELL_ALL_MLL, B-CELL_ALL_T-ALL,B-CELL_ALL_TCF3-PBX1 are represented by 0, 1, 2, 3, 4, 5, 6 respectively.

**Table 11:** Performance of models on original dataset.

Model	Accuracy	Precision	Recall	F1 score
LVTrees	0.91	0.95	0.89	0.89
KNN	0.91	0.95	0.88	0.88
ETC	0.88	0.80	0.84	0.82
ADA	0.65	0.78	0.67	0.67
SVC	0.91	0.96	0.88	0.88
RF	0.88	0.81	0.84	0.82
NB	0.86	0.79	0.81	0.79
DT	0.72	0.74	0.72	0.73
LR	0.91	0.95	0.88	0.88

### Models performance using ADASYN oversampled dataset

**Figure 4:**
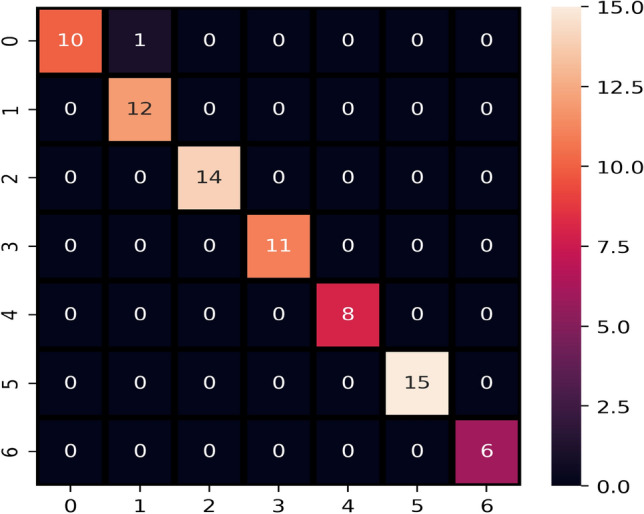
Confusion matrix of best performer LVTrees after applying ADASYN technique.

The performance of models is improved after applying the ADASYN technique because it generates new data to make the dataset balanced for all target classes. Balancing of data increases the dataset’s size which helps to improve the performance of learning models and reduce the chances of model over-fitting. Experimental results after applying the ADASYN technique are shown in Table 12. LR, RF, SVC, and LVTrees equally perform well with a 0.99 accuracy score. ADA performs poorly because the dataset’s size is not enough for the good fit of boosting algorithm. The confusion matrix for the best performer LVTrees is shown in Fig. 4. According to the confusion matrix, LVTrees gives only one wrong prediction out of 77 predictions and gives 76 correct predictions, which shows the significance of LVTrees with the ADASYN technique.

**Table 12:** Performance of models applying ADASYN technique.

Model	Accuracy	Precision	Recall	F1 score
LVTrees	0.99	0.99	0.99	0.99
KNN	0.87	0.91	0.88	0.87
ETC	0.97	0.98	0.98	0.98
ADA	0.75	0.86	0.78	0.77
SVC	0.99	0.99	0.99	0.99
RF	0.99	0.99	0.99	0.99
NB	0.95	0.95	0.95	0.95
DT	0.87	0.87	0.88	0.87
LR	0.99	0.99	0.99	0.99

### Models’ performance after applying Chi2 technique

The performance of the models is also evaluated using the Chi2 approach.

**Figure 5:**
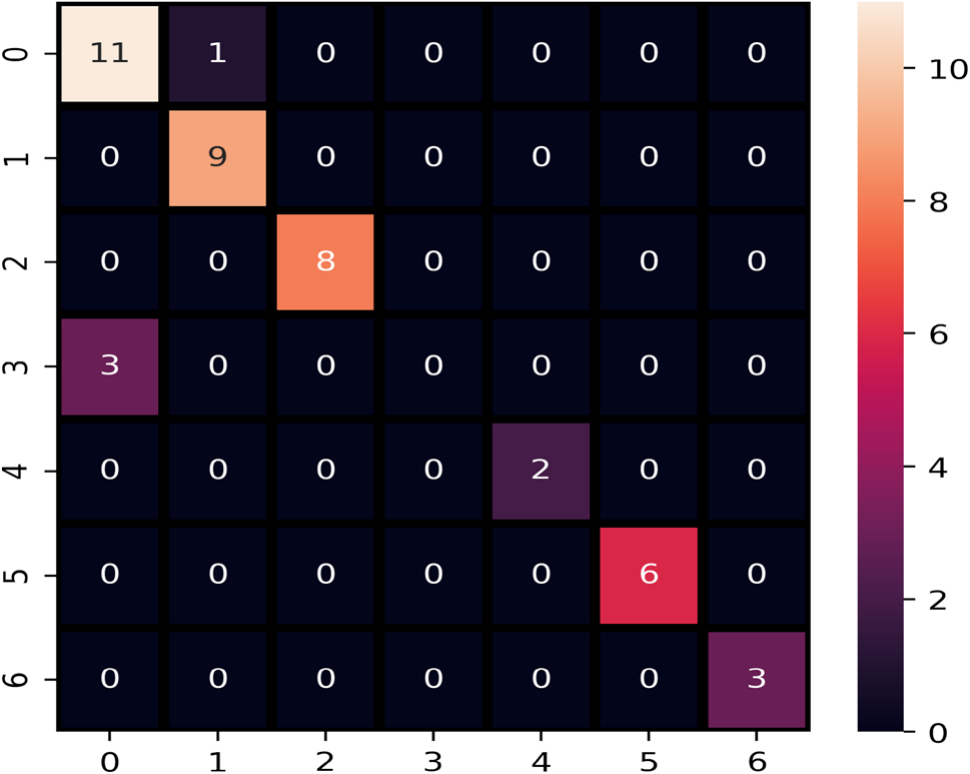
Confusion matrix of LVTrees after applying Chi2 technique.

Results given in Table 13 show that the Chi2 has a slight impact on models’ performance as LVTrees achieves the same accuracy score of 0.91 like it did with the original dataset. Chi2 improves the performance of ADA from 0.65 to 0.72 and DT from 0.72 to 0.74 for the original Chi2 applied dataset. However, the underlying goal is not to compete for the model’s performance with ADASYN. On average, Chi2 improves the performance of models by comparing the performance of models on the original dataset because Chi2 selects only important features for the training of models, which reduces complexity in the training of models and improves the performance. The confusion matrix of best performer LVTrees is shown in Fig. 5 which indicates that LVTrees give 4 wrong predictions out of 43 predictions.

**Table 13:** Performance of models after applying Chi2 technique.

Model	Accuracy	Precision	Recall	F1 score
LVTrees	0.91	0.81	0.85	0.83
KNN	0.79	0.82	0.81	0.81
ETC	0.86	0.79	0.82	0.80
ADA	0.72	0.64	0.59	0.59
SVC	0.86	0.78	0.82	0.80
RF	0.88	0.81	0.83	0.82
NB	0.86	0.85	0.85	0.85
DT	0.74	0.73	0.4	0.73
LR	0.88	0.81	0.83	0.82

### Models performance for combining Chi2 and ADASYN techniques

**Figure 6:**
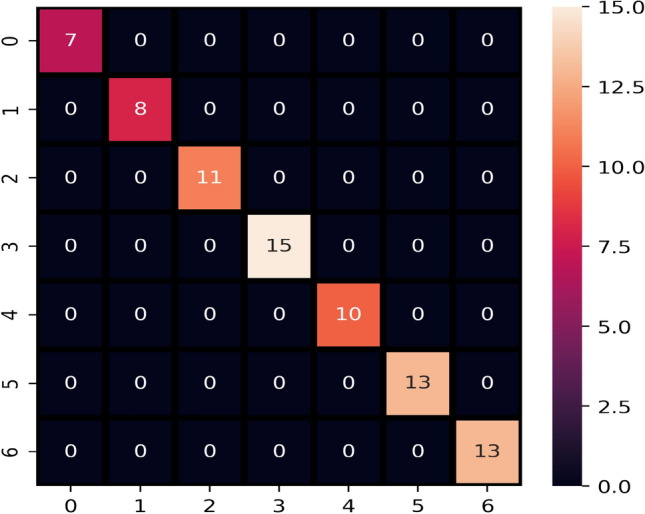
Confusion matrix of LVTrees after applying Chi2 and ADASYN techniques.

**Figure 7:**
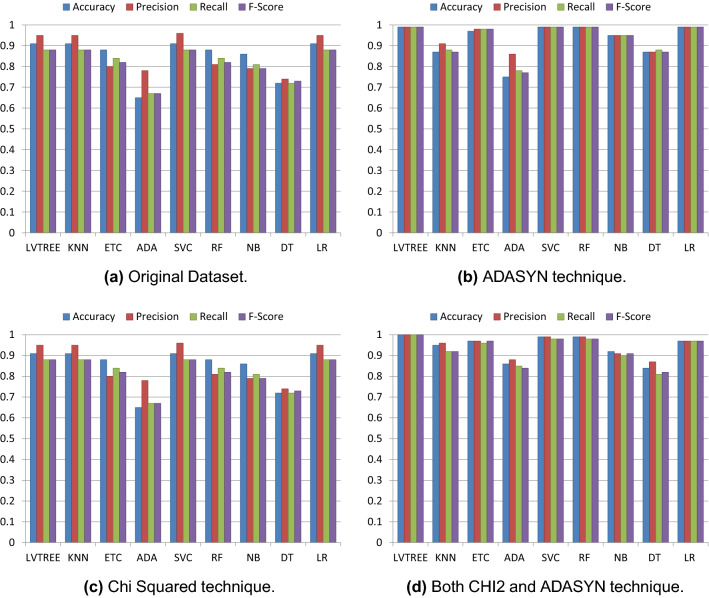
Results of the models’ performance after applying each technique.

**Figure 8:**
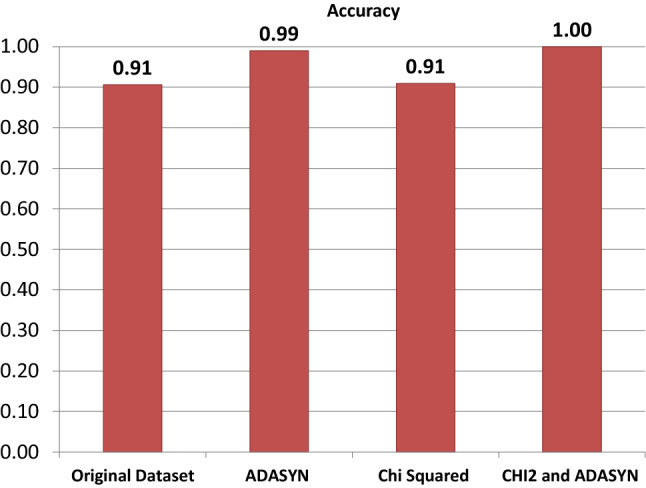
Accuracy score comparison with all approaches.

The ADASYN and Chi2 techniques are combined at the preprocessing and the performance of models is evaluated. Results show significant enhancement when both techniques are combined. Chi2 selects the most significant features concerning the correlation to the target class while ADAYNS help to reduce the probability of model over-fitting for the majority class by generating new data. The performance of the models has been significantly improved when used with ADASYN and Chi2 combined as shown in Table 14. On average all models perform well, however, the proposed model LVTrees outperforms all other models with a 100% accuracy. RF and SVC achieve 0.99 accuracy scores while the accuracy for LR, ETC, KNN is 0.97, 0.97, and 0.95, respectively. The confusion matrix of the LVTrees is displayed in Fig. 6 indicating that LVTrees give 77 correct predictions out of 77 which shows the significance of the proposed approach. Figures 7 and 8 show the performance comparison between all approaches for each case.

**Table 14:** Performance of models after applying both CHI2 and ADASYN techniques.

Model	Accuracy	Precision	Recall	F1 score
LVTrees	1.00	1.00	1.00	1.00
KNN	0.95	0.96	0.92	0.92
ETC	0.97	0.97	0.96	0.97
ADA	0.86	0.88	0.85	0.84
SVC	0.99	0.99	0.98	0.98
RF	0.99	0.99	0.98	0.98
NB	0.92	0.91	0.90	0.91
DT	0.84	0.87	0.81	0.82
LR	0.97	0.97	0.97	0.97

### Significance of proposed approach

To show the significance of the proposed approach, a second dataset has been used for validation. In addition, a statistically significant *T*-test is performed.

#### Experimental results of LVTrees on leukemia_GSE9476 dataset

The performance of the proposed approach is also evaluated on one another dataset to show its validity and significance. Results in Table 15 show the performance of LVTrees with and without our approach on the Leukemia_GSE9476 dataset. LVTrees achieves the highest accuracy score of 1.00 with our approach, while without the proposed approach, it achieved 0.90. On the other hand, tuned machine learning models do not perform well on the Leukemia_GSE9476 dataset.

**Table 15:** Performance of proposed approach on Leukemia_GSE9476 dataset.

Model	Accuracy	Precision	Recall	F1 score
LVTrees	0.90	0.95	0.92	0.92
LVTrees (CHI2+ADASYN)	1.00	1.00	1.00	1.00

### Results using resampling on training data alone

Experiments are performed using the resampling approach on the training data alone. The original dataset is split into training and test sets and resampling is applied only to the training set while the testing is performed with the test set. Resampling on the training data alone is used to avoid any data leakage. Feature selection does not cause any data leakage because it reduces the feature set size and does not impact the number of samples. However, feature selection is to be applied on the test set as well for testing. Experimental results are shown in Table 16. Results show that LVTrees performs well with the resampling approach and obtains better accuracy, recall, and F1 score. The size of testing data is very small without the resampling and the number of samples is different for each class which causes a slight reduction in the classification accuracy.

**Table 16:** Performance of resampling on training data alone.

Model	Accuracy	Precision	Recall	F1 Score
LVTrees (Original)	0.91	0.95	0.89	0.89
LVTrees (Chi+ADASYN)	0.95	0.93	0.95	0.94

### Feature selection after data splitting

For analyzing the significance of LVTrees and avoiding the conflict of a probable data leakage caused by feature selection before data splitting^36^, this study performs feature selection on both train and test sets separately after data splitting. Experimental results of all models are provided in Table 17. Results suggest that the proposed model LVTrees outperform all other models with the highest 0.97 accuracy score. LVTrees is significant in terms of all evaluation parameters while LR, RF, ETC are just behind the LVTrees with a 0.96 accuracy score. These significant results of models obtained with feature selection after data splitting show that the feature selection before or after data splitting does not lead to data leakage.

**Table 17:** Performance results when we done feature selection after data splitting.

Model	Accuracy	Precision	Recall	F1 Score
LVTrees	0.97	0.97	0.97	0.97
KNN	0.89	0.91	0.90	0.89
ETC	0.96	0.96	0.96	0.96
ADA	0.38	0.42	0.45	0.39
SVC	0.95	0.95	0.95	0.95
RF	0.96	0.96	0.96	0.96
NB	0.94	0.94	0.94	0.94
DT	0.86	0.85	0.86	0.85
LR	0.96	0.96	0.96	0.96

### Results with 10-fold cross-validation

This study also performs 10-fold cross-validation to find the mean accuracy and standard deviation (SD) of models’ performance. Cross-validation is applied to show the significance of the proposed approach which comprises Chi2 and ADASYN. The 10-fold cross-validation is carried out directly on the original dataset and after feature selection and data resampling on the dataset. All models improved their accuracy significantly with the proposed methodology. Results for cross-validation are provided in Table 18 which show that LVTrees shows superior performance with a mean accuracy of 0.97 and 0.03 SD. LR, RF, SVC, ETC also have a standard deviation of 0.03 but the mean accuracy is low as compared to LVTrees. ADA has the highest SD value of 0.10 and lowest mean accuracy of 0.57 which is primarily due to the small dataset. Proposed LVTrees is also significant on the original dataset as compared to other models.

**Table 18:** Results of 10-fold cross validation for all models.

Model	Original data		Chi2 +ADASYN	
	Accuracy	SD	Accuracy	SD
LVTrees	0.90	$\pm 0.03$	0.97	$\pm 0.03$
KNN	0.79	$\pm 0.05$	0.92	$\pm 0.04$
ETC	0.86	$\pm 0.03$	0.95	$\pm 0.03$
ADA	0.48	$\pm 0.06$	0.57	$\pm 0.10$
SVC	0.89	$\pm 0.04$	0.96	$\pm 0.03$
RF	0.86	$\pm 0.04$	0.96	$\pm 0.03$
NB	0.83	$\pm 0.07$	0.90	$\pm 0.04$
DT	0.70	$\pm 0.06$	0.86	$\pm 0.05$
LR	0.89	$\pm 0.03$	0.95	$\pm 0.03$

#### Performance analysis of proposed approach

For validating the efficacy and dominance of the proposed approach, its performance is compared with several state-of-the-art approaches, and results are provided in Table 19.

**Table 19:** Comparison with previous approaches.

Reference	Year	Model	Data	Accuracy
^19^	2019	SVM, KNN, NB, and RF	Microarray gene	KNN: 96.28%, NB: 97.29%, RF: 97.01%
^20^	2020	DNNs deep learning network	Microarray gene	96.6%
Current study	2021	LVTrees	Microarray gene	100%

These studies have been selected concerning the fact that they used the same dataset for the experiments. For example, Castillo et al.^19^ perform experiments using various machine learning models with RF showing a 97.28% accuracy. Similarly, Nazari et al.^20^ used a deep learning approach for blood cancer prediction and achieved 96.6% accuracy. In comparison with these studies, our approach achieved 100% of accuracy on the same dataset which shows the significance of the proposed approach.

#### *T*-test

To show the significance of the proposed approach, a *T*-test is performed. The *T*-test is a statistical test used to verify a hypothesis about the performance of a model. It gives results in the form of acceptance and rejection of the null hypothesis. We have a null hypothesis as:

- **Null Hypothesis:** Proposed approach is statistically significant in comparison with other methods.
- **Alternative Hypothesis:** Proposed approach is not statistically significant in comparison with other methods.

*T*-test accepted the null hypothesis when LVTrees is used with ADASYSN and ADASYN+Chi2 compared to original data, which means that the proposed LVTrees is statistically significant in both cases when used with ADASYN and a combination of both ADASYN and Chi2. LVTrees rejected the null hypothesis when LVTrees is used with Chi2, which means that LVTrees is not statistically significant compared to when LVTrees is applied to the original dataset. Besides, LVTrees accept the null hypothesis compared to other used models in all cases, which shows the significance of the proposed model.

## Conclusion

The current study aims at predicting leukemia type of blood cancer using an imbalanced dataset. Despite several approaches, accuracy using imbalanced and high dimensional datasets remain an open and challenging endeavor. To this end, a hybrid of machine learning models, called LVTrees, is proposed which uses leukemia microarray gene data for experiments. On account of the imbalance dataset, ADASYN oversampling approach is adopted while Chi2 is used to eliminate the features that have little correlation to the target class thus resolving the problem of high dimension. Extensive experiments are performed using ADASYN and Chi2 approaches alone, as well as, the proposed approach where both approaches are combined. ADASYN tends to improve the performance of both machine learning models and proposed LVTrees. Oversampling helps to reduce the class difference and boosts the performance by reducing the probability of the model overfitting. Conversely, Chi2 shows a slight impact on the prediction accuracy. Although Chi2 reduces complexity by selecting only the best features for model training, not all the models show performance improvement. In the proposed approach where both ADASYN and Chi2 are used together, LVTrees show superior performance and achieve 100% cancer prediction accuracy. LVTrees hybrid architecture used these best features to get a good fit and make predictions under majority voting criteria. In addition, the validation results on the second dataset and performance comparison with state-of-the-art approaches show the validity and dominance of the proposed approach. Results suggest that a combination of multiple models could perform well as compared to an individual model. For the future, a customized deep learning model which could perform well on small datasets is envisioned. In addition, combining multiple datasets to make a complicated and high dimensional dataset for conducting experiments with the proposed approach is under consideration.
